# To what extent are objectively measured mammographic imaging techniques associated with compression outcomes

**DOI:** 10.1259/bjr.20230089

**Published:** 2023-04-20

**Authors:** Sue M Hudson, Louise S Wilkinson, Bianca L De Stavola, Isabel dos-Santos-Silva

**Affiliations:** 1 Department of Non-Communicable Disease Epidemiology, London School of Hygiene and Tropical Medicine, Keppel, London, UK; 2 Oxford Breast Imaging Centre, Oxford University Hospitals NHS Foundation Trust, Oxford, UK

## Abstract

**Objective:**

To describe the association between objectively measurable imaging techniques and the resulting compression thickness and dose.

**Methods:**

The study included 80,495 routine screens from the South-West London Breast Screening Service between March 2013 and July 2017. Average compression force, paddle tilt and dose were calculated. The Volpara^®^ DensityTM algorithm was used to estimate pressure, breast volume and density.

Linear regression models, using generalized estimating equations (GEEs) to account for clustering by practitioner, assessed the strength of the associations between the imaging compression outcomes, (thickness, dose) and imaging techniques (force, pressure and paddle tilt), adjusting for the subject’s characteristics (age, ethnicity, breast volume and percent mammographic density).

**Results:**

Fully adjusted linear regression models showed that compression thickness decreased by ~1 mm (~2% of mean thickness) for every 1daN increase in force and decreased by ~0.8 mm with an increase of 1 kPa of pressure (at median pressure). Increasing pressure above 15 kPa resulted in minimal reduction in thickness. Dose increased with increased force but decreased by ~1% of mean dose with every increase in 1 kPa of pressure. For 1^o^ increase in paddle tilt, the compression thickness increased by ~1.5 mm (~2.5%) and dose increased by ~2.5%, (Pt <0.001 in all cases).

**Conclusion:**

Differences in imaging technique are associated with imaging outcome measures (thickness and dose). A better understanding of the association between objective image acquisition parameters and tumour conspicuity could lead to clearer guidelines for practitioners.

**Advances in knowledge:**

Increased paddle tilt is associated with increased compression thickness and increased dose after adjustment for breast volume and force applied.

## Introduction

Breast cancer screening, through mammography, offers one approach to reducing mortality from breast cancer through early diagnosis of non-palpable tumours followed by early treatment. In England and Wales, ~3 million women, aged 50–70 years are invited once every 3 years to undergo standard 2-view mammography of each breast as part of the National Health Breast Screening Programme (NHSBSP).^1^ Although the radiation risks associated with screening mammography are relatively low, the regular exposure of well-women to potentially harmful X-ray exposures should be kept to the minimum required to obtain an adequate breast image.^2^ Not all mammographic images however are high quality and any factor that leads to a reduction in image quality could be detrimental to cancer detection. A key factor that influences both absorbed dose and image quality, is breast compression. Compression is required to reduce movement, separate superimposed tissue and to reduce tissue thickness which is associated with both increased tumour conspicuity^3,4^ and reduction in radiation load.^5,6^ At the same time, breast compression is associated with pain which may deter females from attending a routine screen.^7,8^ There is evidence that ‘too much’ compression can lead to unnecessary pain for no additional improvement in image quality,^9,10^ however, research remains very limited in this area.

Despite the importance of compression technique, guidelines remain largely subjective and the most recent NHSBSP recommendations, which stipulate regular image quality audits, are restricted to subjective checks for image sharpness and for ‘adequate compression to hold breast firmly/no movement’.^6^ Earlier NHSBSP guidelines, in force at the time images in this study were taken, also included the recommendation that force should not exceed 20 daN.^11^

**Figure 1.**
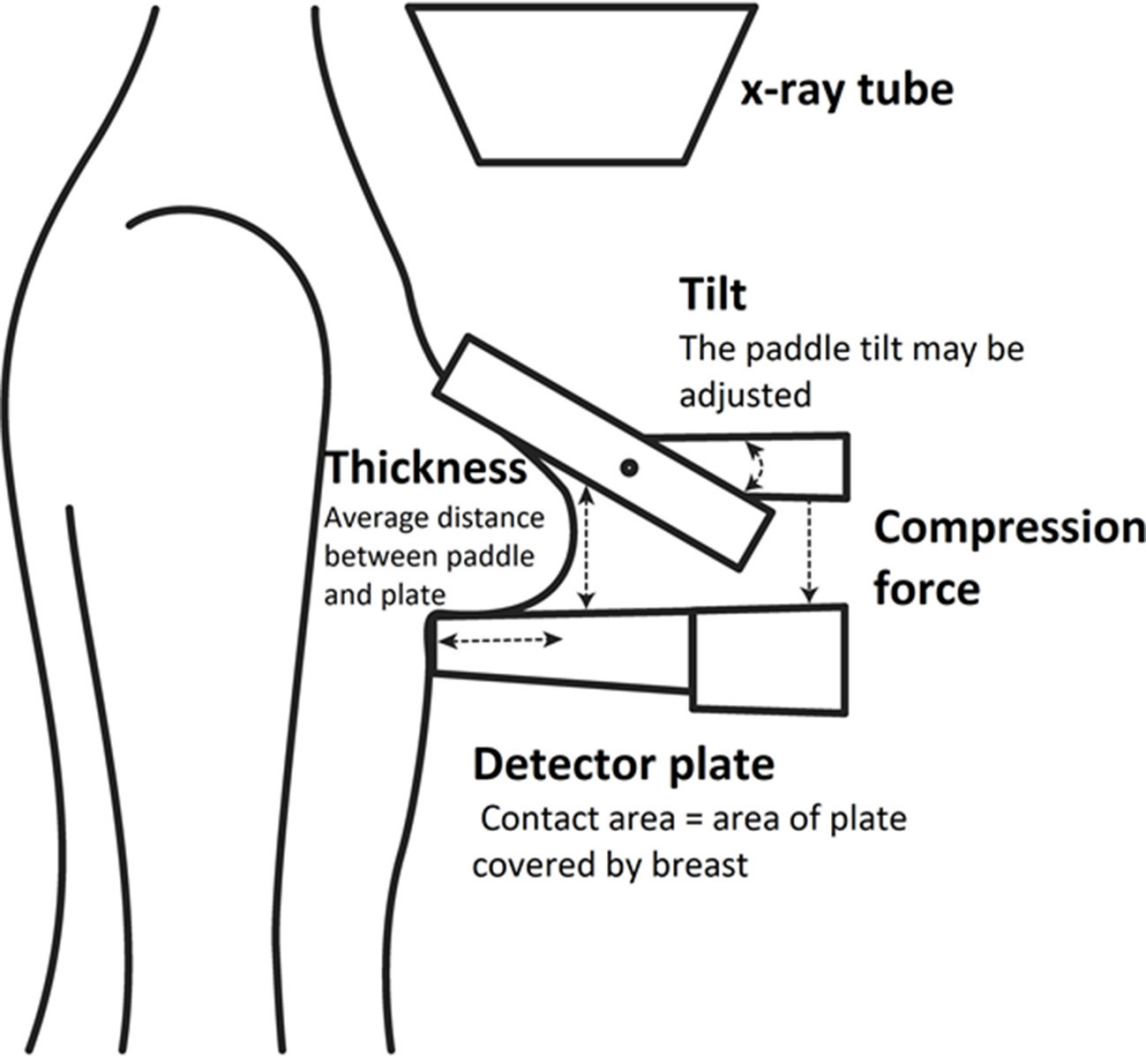
Compression of the breast during CC image acquisition schematic. CC, craniocaudal.

Compression is achieved by pressing the breast against the top of the detector using a transparent plastic compression paddle (Figure 1). The force applied can be monitored by the practitioner during the process however ‘pressure’, (force divided by the area of contact between breast and detector plate) may be better measure of compression, because it takes account of the size of the breast as well as the force applied.^12^ Studies show that screening performance, including cancer detection, may be associated with compression pressure.^13,14^ With a rigid paddle, the paddle remains parallel to the detector during imaging. The optional, flexible paddle, was introduced by equipment manufacturers to make the process of mammography more comfortable, allowing the practitioner to ‘tilt’ the paddle using a hinging mechanism to accommodate the shape and size of the breast, although the effectiveness of these flexible paddles for pain reduction has been queried^15,16^ as has their effect on image quality and dose.^15,17^

Studies have found considerable variation in breast compression force and pressure between practitioners^18–20^ both within screening organisations^21,22^ and also across different countries.^23,24^ Studies have also shown that the breast characteristics such as mammographic density are correlated with compression force, pressure and thickness.^25^ Less is known about the variation in flexible-paddle tilt, used during image acquisition and its association with compression measurements. It is also possible that the ethnicity of the subject being screened could modify any association between compression parameters and compression outcomes, if, *e.g.* slight physiological differences between ethnic groups influenced the way that mammography is conducted, likewise the actual mammography machine may modify these associations due to physical location and constraints.

Mammographic screening in the UK utilises full-field digital mammography and the resulting images may be processed using automated algorithms which provide comprehensive image acquisition data as well as volumetric estimates of both breast size and mammographic density. Therefore, it has become feasible to carry out large-scale studies based on objective image acquisition parameters.

This study aims to describe the variation in the image acquisition parameters that are controlled by the practitioner during the imaging process (*i.e.* force, pressure and paddle tilt) using a sample of over 80,000 examinations of females who participated in a population-based breast screening programme. We believe that this will be the first large-scale study to include the variation in use of the flexible paddle. Secondly, the study aims to describe the association between imaging technique (force, pressure and paddle tilt) and the resulting compression thickness and dose (after adjusting for the screening subject’s breast volume and density) since research suggests that these factors are likely to be key to successful diagnostic imaging in a screening programme.

## Methods

### Study participants

The study includes mammographic examinations undertaken in the period 01 March 2013 to 20 June 2017 as part of the NHSBSP routine 3-yearly screening programme at the South*-*West London Breast Screening Service (SWLBSS) based in the St George’s University Hospitals National Health Service (NHS) Foundation Trust. Females screened were resident in one of six London boroughs—Wandsworth, Merton, Croydon, Sutton, Richmond and Kingston and all were asymptomatic for breast cancer at the time of screening. Data on ethnicity were recorded according to the Census classification^26^ as part of standard practice via a self-completed screening questionnaire. The practitioner responsible for each screening examination (*i.e.* each set of four images) was recorded on the screening administrative system.

Each screening examination consisted of the NHSBSP standard 2-view [craniocaudal (CC) and mediolateral oblique (MLO) views] mammography of each breast.^27^ The raw anonymised digital mammographic images were processed using the automated algorithm, Volpara^®^ Density™ v. 1.5.11 (Matakina Technology Limited, Wellington, New Zealand)^28^ to generate automated estimates (in cm^3^) of the volume of the breast (BV) and the volume of the radiodense tissue (DV) for each image. Mammographic density (%MD) was estimated as DV/BV×100. Data on the imaging technique were also collected; compression force (decaNewton, daN), compressed breast thickness (mm) and compression paddle tilt (degrees from horizontal) which were available from the Digital Imaging and Communications in Medicine (DICOM) image header. The Volpara^®^ Density™ algorithm estimated contact area (cm^2^) between breast and plate for each image and the resulting pressure (kiloPascals,kPa) from force*10/contact area. The mean glandular dose (MGD)(in milli Gray (mGy)) as calculated by the machine manufacturer and the identification of the mammography machine ‘detector’ were available from the DICOM header.

### Exclusions

In all, 94,408 screening examinations were carried out during the study period. We excluded examinations for which no reason was specified (*i.e.* screening episode type missing) (*n* = 992). Examinations were also excluded if there were not exactly four images taken, because the automated algorithm is not designed to make estimates in these circumstances.^28^ Thus, we excluded examinations of females who have exceptionally large breasts requiring additional (mosaic) images, examinations that were repeated for technical reasons, and examinations where fewer images were taken because of mastectomy or lack of tolerance of the procedure (*n* = 10,882; Supplementary Table 1). Because of potential differences between manufacturers, examinations using non-Hologic systems were also excluded (*n* = 626) and we also excluded examinations on subjects known to have previous breast cancer (*n* = 1413) because this may influence the imaging technique, leaving a total of 80,495 examinations (321,980 compressions) eligible for inclusion in the analysis (Supplementary Figure 1).

Supplementary Table 1.

Supplementary Figure 1.

### Ethical approval

This retrospective study was carried out on fully anonymous, routinely collected data only, held in accordance with the NHS Cancer Screening Programmes Confidentiality and Disclosure Policy 2011. The NHSBSP has section 251 support under the NHS Act 2006. The study was approved by all relevant ethics committees (Research Ethics Committees from St George’s University Hospitals NHS Foundation Trust, and the London School of Hygiene and Tropical Medicine).

### Statistical methods

The distributions of the imaging parameters (force, pressure and paddle tilt) and the imaging compression outcomes (dose and compressed breast thickness) were examined. Scatter plots were created to examine the distribution and Spearman correlations between the outcomes and imaging parameters. Similarly, scatter plots and Spearman correlation coefficients were used to examine the correlations between imaging compression outcomes (thickness, dose) and the characteristics of the imaging subject (age, BV, %MD). A line of best fit was calculated for each plot using a locally weighted scatterplot smoothing (Lowess) function.

Linear regression models were used to examine the strength of the associations between the imaging outcomes, (thickness and dose) and the three imaging parameters (force, pressure and paddle tilt, treated as continuous variables), after adjusting for the subject’s characteristics (age, BV and %MD). Spearman rank correlation coefficients were used to identify potential collinearity between predictors in the proposed models. Models for each exposure were further adjusted for the other two compression parameters where collinearity was not an issue. General estimating equations (GEEs) and robust standard errors (clustering by practitioner) were used to account for the fact that each practitioner carried out multiple examinations in the study period and practitioners may have their own imaging technique. Tests for departure from linear trend were conducted by including quadratic terms for each exposure variable and plotting the estimated exposure response curves. In all the analyses, regression coefficients represent the change in per one-unit change in the exposure variable.

We categorised compression parameters (force, pressure and tilt) into high and low categories and used linear regression models to test for effect modification by ethnicity or mammography machine (detector) on the association between compression parameters and outcomes (thickness and dose).

We considered statistical significance (two-sided) at *p*-value < 0.05. All analyses were conducted in Stata (IC 14).^29^

## Results

### Characteristics of screening examinations

The characteristics of the 80,495 screening examinations are shown in Table 1. The majority (~86%) of examinations were on females who were within the ages of 50–70 years, the main age-group targeted by the NHSBSP. Among the 86% of the subjects who reported their ethnicity, ~76% were White but there were also high numbers of females of Black and Asian ethnicity. In all, 87 different practitioners carried out examinations during the study.

**Table 1.** Technical and subject characteristics associated with screening examinations

	**Frequency**	**Percent %**
**All standard screening examinations^a^**	80,495	
**Age at screening, years**		
<45-	551	0.70%
45–49	5,928	7.40%
50–54	22,082	27.40%
55–59	18,175	22.60%
60–64	15,080	18.70%
65–69	13,633	16.90%
70+	5,046	6.30%
Missing	0	0.00%
**Ethnicity (of subject screened^b^**)		
White—British or Irish or other	52,461	65.20%
Asian—British Indian or Pakistani or Bangladeshi or other	7,611	9.50%
Black—British or Caribbean or other	3,745	4.70%
Black—African	2,725	3.40%
Mixed White and Black, White and Asian or any other mixed	1,526	1.90%
Chinese	1,062	1.30%
Missing or not reported	11,365	14.10%
**Breast volumetric measurements^c^**	**Median**	**IQR**
Breast volume, cm^3^	753	489–1,110
Breast dense volume, cm^3^	49.4	37.2–67.3
Mammographic density, %	6.40%	4.6–10.2%
**Imaging acquisition parameters average across MLO and CC views^c^**	**Mean**	**SD**
Mean compression force applied, daN	8.27	2.09
Mean paddle tilt angle, degrees positive from horizontal	2.69	1.06
Mean pressure, kPa		
**Imaging outcome estimates average across MLO and CC views^c^**	8.6	3.53
Manufacturers mean glandular dose, mGy^d^	1.32	0.36
Mean breast thickness, mm	56	12.4
**Imaging acquisition parameters for MLO^e^**	**Mean**	**SD**
Mean compression force applied, daN	9.12	2.59
Mean paddle tilt angle, degrees positive from horizontal	2.87	1.28
Mean pressure, kPa	7.36	2.51
**Imaging outcome estimates for MLO^e^**	1.39	0.41
Mean glandular dose, mGy^d^	57.8	13.67
Mean breast thickness, mm		
**Imaging acquisition parameters for CC^e^**	**Mean**	**SD**
Mean compression force applied, daN	7.74	1.95
Mean paddle tilt angle, degrees positive from horizontal	2.43	1.16
Mean pressure, kPa	9.97	4.96
**Imaging outcome estimates for CC^e^**		
Mean glandular dose, mGy^d^	1.25	0.34
Mean breast thickness, mm	54.08	11.72

CC, craniocaudal;DICOM, Digital Imaging and Communications in Medicine; IQR, interquartile range; MLO, mediolateral oblique; SD, standard deviation.

a A screening examination was included if it had exactly four images taken, only screening appointments were included. We excluded images taken on non-Hologic systems and screens where females were known to have previous cancer. Total number of images (compressions) was 321,980

b Data on ethnicity were collected as part of standard screening protocol via a self-completed screening questionnaire and recorded according to the Census classificationand summarised as, “Asian” (Indian, Pakistani or Bangladeshi or other), “Black-African”, “Black-British or Caribbean or other”, “Chinese”, “Mixed” (White and Black, White and Asian or any other mixed), “White” (British or Irish or other) and “Other”. Count per screening examination (subjects may have more than one examination over the study period).

c Breast volumetric measures were calculated from the mean value from the four images: left CC image, right CC image, left MLO image, right MLO image.

d Manufacturers mean glandular dose as recorded in DICOM header.

e Calculated from the average value from the two relevant images left and right sides.

The mean force applied during a single MLO compression was higher than that for a CC compression (9.12 and 7.74 daN respectively), likewise, the mean paddle tilt was greater during an MLO compression than a CC compression (2.87^o^ and 2.43^o^ from horizontal respectively). In contrast, the mean pressure was higher for CC views than for MLO views (9.97 and 7.36 kPa respectively). The mean of MLO and CC values were used for this study unless otherwise stated.

The distributions of the imaging parameters and the outcomes were approximately normal (Supplementary Figure 2).

Supplementary Figure 2.

### Correlations between characteristics of screening subject and compression outcomes

**Figure 2.**
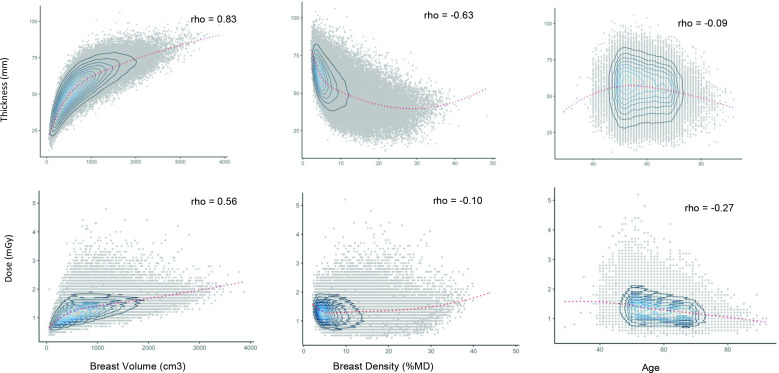
Scatter plots and heat maps of compression outcomes (thickness and dose) against characteristics of imaging subject (lowess smoothing). All measurements derived from average of four mammographic views. Left breast CC, Right breast CC, Left breast MLO, Right breast MLO. Dose is manufacturer’s recorded mean glandular dose in milligray (mGy). Showing Spearman’s correlation coefficient (ρ). CC, craniocaudal; MLO, mediolateral oblique.

There was a strong/moderate correlation between the compression outcomes and the subject’s BV with larger BV associated with increased thickness and dose (Spearman correlation coefficient (ρ): 0.83 and 0.56, respectively; *p* < 0.001 for both). Both compression thickness and dose were negatively correlated with %MD (ρ = −0.63 and −0.11, respectively; *p* < 0.001 for both). The compression outcomes (thickness and dose) both decline somewhat with age of subject, but the correlations were very weak (Figure 2).

### Associations between imaging parameters and compression outcomes

**Figure 3.**
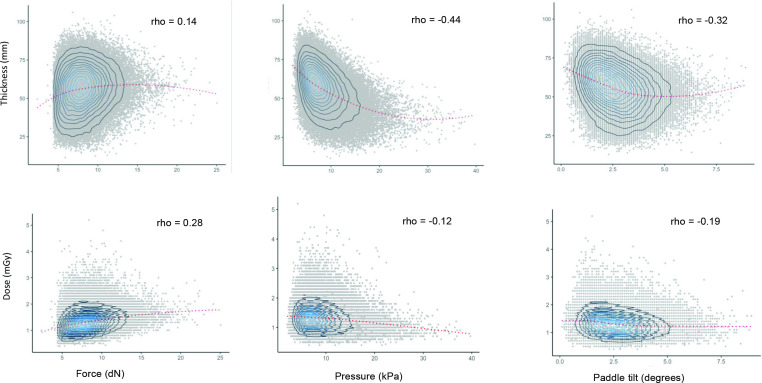
Scatter plots of compression outcomes (thickness and dose) with image acquisition parameters (force, pressure and paddle tilt) lowess smoothing. All measurements derived from average of four mammographic views. Left breast CC, Right breast CC, Left breast MLO, Right breast MLO. Dose is manufacturer’s recorded mean glandular dose in milligray (mGy). Showing Spearman’s ρ correlation coefficient. Tilt is positive tilt in degrees from horizontal. CC, craniocaudal; MLO, mediolateral oblique.

Figure 3 shows weak positive Spearman’s correlations between force and the outcome measures (thickness ρ = 0.14; and dose ρ = 0.28; *p* < 0.001 in both cases). In contrast the correlation between pressure and thickness is moderate but is negative (ρ = −0.44, *p* < 0.0001) and the relationship appears to be non-linear.

The adjusted fitted linear regression model shows that compression thickness decreased with increasing force (P for trend (Pt) <0.001; Table 2), after a simple adjustment for BV alone. Further adjustment for %MD, age and paddle tilt did not change the direction of the association, but the regression coefficients were strengthened somewhat (β = −0.81 and −1.07, respectively; *p* < 0.001 for both). There was little evidence for departure from linear trend in the exposure response plots (Supplementary Figure 3).

Supplementary Figure 3.

**Table 2.** Linear regression analysis of associations between compression parameters (force, pressure and tilt) and compression outcomes (thickness and dose) crude and after adjustment ^a^

	**Compression thickness mm(*n* = 79,476**)			
	**Crude**	**Adjusted for breast volume**	**Fully adjusted^a^**	**Mutually adjusted^b^**
	**Change per unit (95% CI)**	**Change per unit (95% CI)**	**Change per unit (95% CI)**	**Change per unit (95% CI)**
**Force daN**	1.89 (1.0, 2.19)	−0.81 (-0.97,–0.65)	−0.92 (-1.07,–0.77)	−1.07 (-1.21,–0.92)
**Pressure kPa**	−1.52 (-1.60,–1.43)	N/A^c^	−0.79 (-0.85,–0.73)	−0.59 (-0.65,–0.53)
**Paddle tilt ^o^**	−3.25 (-3.43,–3.07)	1.48 (1.36, 1.60)	1.27 (1.15, 1.39)	1.48 (1.36, 1.59)
	**Mean glandular dose milliGray^d^ (*n* = 69,924)**			
	**Crude**	**Adjusted for breast volume**	**Fully adjusted^a^**	**Mutually adjusted^b^**
	**Change per unit (95% CI)**	**Change per unit (95% CI)**	**Change per unit (95% CI)**	**Change per unit (95% CI)**
				
**Force daN**	0.061 (0.056, 0.067)	0.017 (0.013, 0.021)	0.023 (0.019, 0.028)	0.020 (0.016, 0.024)
				
**Pressure kPa**	−0.015 (-0.016,–0.013)	N/A^c^	−0.019 (-0.021,–0.017)	−0.011 (-0.131,–0.009)
**Paddle Tilt ^o^**	−0.060 (-0.064,–0.057)	0.029 (0.021, 0.035)	0.041 (0.036, 0.046)	0.037 (0.032, 0.042)

BV, breast volume ; CI, confidence interval; GEE, generalized estimating equation.

P for linear trend <0.001 in all cases

a Adjusted for: %MD, Age with GEE and robust standard errors to account for mammographer clusters. Force and Tilt models additionally adjusted for BV, which was omitted in the Pressure model due to collinearity.

b Mutually adjusted, *i.e* force models additionally adjusted for tilt;pressure models additionally adjusted for tilt; tilt models additionally adjusted for force. Pressure omitted from force and tilt models due to collinearity.

c Pressure models are not additionally adjusted for breast volume due to collinearity.

d Manufacturer’s estimatedmean glandular dose.

Thickness also decreased with increasing pressure, (Pt <0.001; Table 2) when controlling for %MD, age and paddle tilt. BV adjustment was omitted from the pressure model due to collinearity because it is strongly negatively correlated with pressure (ρ = −0.73 see Supplementary Table 2). The exposure response curve shows that, at increased pressures, there was no longer a reduction of breast thickness suggesting a diminishing return from additional pressure above an optimal point, after controlling for %MD, age and paddle tilt (Supplementary Figure 3).

Dose increased with increasing force (Pt<0.001 in all models; Table 2) although this was attenuated after adjustment for BV. In contrast dose decreased slightly with increasing pressure (Pt<0.001) after adjustment for %MD, age and paddle tilt.

Both thickness and dose are weakly negatively correlated with paddle tilt (ρ = −0.32 and −0.19, respectively; *p* < 0.001 for both), suggesting that, in a model where there is no adjustment for breast volume, both dose and thickness decline with increasing paddle tilt. However, after controlling for BV, %MD, age and force, compression thickness increased with increasing paddle tilt and there was no evidence of departure from linear trend(Pt<0.001; Table 2)). For each 1^o^ increase in paddle tilt, the compression thickness increased by ~1.5 mm (~2.5%). Likewise, after adjustment for BV and force, we found a positive association between dose and paddle tilt. For each 1^o^ increase in paddle tilt, dose increased by ~2.5%, (P for trend (Pt) <0.001; Table 2) in the fully adjusted model.

There was evidence for an interaction between ethnicity and force applied (*p* < 0.001; results not shown) after adjustment for BV, %MD and age. However, the differences in coefficients between the different ethnicities represented small differences in thickness (~1 mm).There was no evidence of interaction between detector plate and explanatory variables in any of the models.

## Discussion

### Main findings

Our study of over 80,000 screening examinations shows that there is large variation in imaging technique, as measured by the compression parameters; force, paddle tilt and pressure. Although the strongest correlate of compression outcome measures (thickness and dose) is BV these outcomes were also associated with the technique applied after adjustment for BV. Compression thickness decreased by ~1 mm (~2% of mean thickness) for every 1 daN increase in force after adjusting for the imaging and subject dependent confounders (tilt, BV, %MD, and age). Thickness decreased by ~0.8 mm (~1.5% of mean thickness) with an increase of 1 kPa of pressure after adjustment for tilt, %MD and age (quadratic model at median pressure value). Dose increased by 1.5% of mean dose with 1 daN increased force (after adjustment), whereas dose decreased by ~0.8% of MGD with every increase in 1 kPa of pressure after adjustment. Outcome measures were also associated with the degree of paddle tilt employed, after full adjustment for subject-dependent confounders. For every 1^o^ increase in paddle tilt, the compression thickness increased by ~1.5 mm (~2.5%) and dose increased by 0.037 mGy (~2.5%). This supports findings in a Dutch study, which found that mean radiation dose was 4.5% lower when rigid (horizontal) paddles were used rather than tilting paddles.^15^

Whether these changes are of clinical relevance is uncertain, although Salvagnini found that lesion detectability decreased from 70 to 37% as thickness increased from the lowest thickness quartile group (<29 mm) to the greatest thickness quartile (>70 mm), in a study of simulated lesions in real breast images.^3^

As expected, compression outcomes were correlated with the characteristics of the subjects being screened; a larger BV was strongly correlated with increased thickness and moderately correlated with higher dose. Thickness was negatively correlated with %MD (possibly because higher %MD is associated with smaller BV); a finding that is similar to Khan-Perez et al in a study of 211 UK females^30^ and Waade from a study of ~11,000 women in Norway.^31^ We found a low correlation between dose and %MD, a finding also supported by Khan-Perez et al.^30^ Ng et al analysed images from 17 different counties and concluded that beyond the practitioner’s breast compression choices, the subject’s age, and breast composition (BV and %MD), there are other factors influencing compression.^24^

Overall mean force and pressure were low in our study in comparison with a study of ~37,000, similar-aged, Dutch females, using the same analytical algorithm^23^; mean MLO force in the Netherlands was 13.8 (SD 2.7) daN compared to a mean of 9.12daN (SD 2.59) in our study. The practitioners in the Netherlands used protocols instructing them to compress to at least 12 daN but at the time that our study data were collected, the UK NHSBSP guidelines did not specify a minimum compression force and were limited to the guidance that force should not exceed 20 daN.^11^ Mean MLO pressure (Netherlands) was 13.7 kPa (SD 5.9) compared to 7.36 kPa (SD 2.51) in our study. Comparative results from the USA^23^ were 7.4 daN (SD 3.1) for force and 8.1 kPa (SD 4.1) for pressure, similar to our study. A Norwegian study on ~18,000 examinations found large variation between centres and reported a mean force (average of CC and MLO compressions) of 11.6 daN; higher than our mean of 8.27 daN.^32^ This suggests that even across European screening programmes where guidelines^5^ have been shared, there is a large variation between programmes. In our study, mean tilt was 2.69^o^ (SD = 1.06; range 2.29 to 3.15), somewhat lower than the 3.73 ^o^ (SD = 2.18) reported by Kallenburg et al. from a sample of 287 examinations in Netherlands that used flexible paddles.^33^ Note, however, that the mean resulting thickness achieved in our study (57.80 mm) was lower than, but very similar to both the Netherlands and US studies (60.7 mm and 59.9 mm respectively) suggesting that the direct comparisons of compression force across different screening populations are not straightforward.

### Strengths and limitations

Strengths of this study include its population-based design and large sample size. We believe that this is the first large study to look at the association of paddle tilt with compression outcomes in a large screening population.

A limitation of this study was that most females were post-menopausal, and average breast density is likely to be lower than a sample that includes younger females, therefore findings are only applicable to this age range. A large number (10,212) of females undergoing routine screening were excluded from the study because there were not exactly four images taken. Whilst the demographic characteristics of the excluded group are not very different from the main study group, it would still be informative to study this group using other technology. We only included examinations from a single breast screening unit where we might expect some consistency due to local quality assurance and supervision. We used one specific algorithm for estimating breast measurements, however this algorithm has been found to produce reliable and repeatable results.^34–36^ The study also uses the X-ray machine manufacturers’ own estimate of MGD, which has been shown to be rather a crude estimate^37^ and not specifically adapted to incorporate tilting paddles. None-the-less despite these uncertainties, the general findings related to dose are likely to be of interest.

### Implications

Compression outcomes are important because Salvagnini et al^3^ have shown, using simulated breast lesions, that tumour detectability increases with reduced compressed breast thickness. Furthermore. recent studies in both the Netherlands^14,38^ and Norway have found that cancer detection is associated with force and pressure used in the image acquisition process. Alongside these considerations, the breast is a radiation-sensitive organ and it is important restrict the glandular dose as much as possible without compromising image quality and cancer detection.

However, these relationships are complex and in our study, we found that increasing pressure beyond 15 kPa, had substantially diminishing returns in terms of decreased thickness, supporting the suggestion from Hogg et al. that, above a certain level (~13 daN in their UK study on younger, symptomatic females), increased force does not reduce thickness considerably and could be avoided,^39^ although their study is not directly comparable.

Flexible (tilting) paddles were introduced as a way of reducing pain during mammography but Broeders et al^15^ and Moshina et al^16^ found no pain reduction during use. Because increased paddle tilt is associated with increased compression thickness and dose after adjustment for BV, it is possible that flexible paddle use has a detrimental effect on screening performance without any reduction in pain. Further studies are required to examine the association between flexible paddle use and breast cancer detection.

Our findings suggests that compression force, pressure and tilt are not systematically adjusted in accordance with subjective breast characteristics and consequently, there is inconsistency in technique and outcome. In particular, force is not systematically adjusted to reflect BV, resulting in variation in pressure, with larger females being compressed using lower pressures and smaller females experiencing higher pressures. Our study further suggests that ethnicity may play a role in the imaging process, but further research is required in this area.

Studies by de Groot et al. in the Netherlands also found that females with smaller BV experienced severe pain more commonly than other subjects^40^ suggesting that protocols are not always appropriate for females of smaller BV. They proposed that pressure-based guidelines could be better than force-based guidelines in mammography. Our study suggests that force-based guidelines could be appropriate but only if controlled for breast volume. However, under real-time conditions, objective measures of BV are not available and therefore pressure guidance may provide a practical alternative. A recent systematic review by Serwan et al, looked at the relative merits of introducing a pressure-standardised protocol in place of force standardisation and concluded that pressure-standardised protocols could be implemented to reduce pain levels without compromising image quality.^41^ Until recently, real-time estimates of detector plate contact area were not readily available, which made real-time estimation of pressure impossible and hence implementation of pressure protocols was impractical in a screening setting. However, recent technological developments are becoming available to support the introduction of such protocols.

Mammography involves consideration of both objective and subjective parameters and an ‘appropriate pressure’ level is achieved using judgement about size, density and elasticity of the subject’s breast as well as the subject’s pain tolerance. It is possible that a better understanding of the association between directly measurable image acquisition parameters and tumour conspicuity could add to this judgement and inform new guidelines, potentially improving overall screening performance through the provision of more objective imaging guidelines.
