# Supplementary material for: To what extent are objectively measured mammographic imaging techniques associated with compression outcomes
Source: Br J Radiol. 2023 Apr 20;96(1146):20230089. doi: 10.1259/bjr.20230089 (PMC10230394; doi:10.1259/bjr.20230089)
Supplement: Supplementary Table 1. [file bjr.20230089.suppl-04.docx]

**Supplementary Table 1** **Characteristics associated with screening examinations in non-standard image sets**

|  | **Frequency** | **Percent %** |
| --- | --- | --- |
| **All non-standard screening examinations** ^a^ | 10,212 |  |
| **No of images taken** |  |  |
| <2 | 50 | 0.49% |
| 2 | 6,299 | 61.68% |
| 3 | 362 | 3.54% |
| 5 | 40 | 0.39% |
| 6 | 652 | 6.38% |
| 7 | 9 | 0.09% |
| 8 | 2,396 | 23.46% |
| >8 | 404 | 3.96% |
|  |  |  |
| **Age at screening** |  |  |
| <45- | 81 | 0.79% |
| 45-49 | 404 | 3.96% |
| 50-54 | 2,758 | 27.01% |
| 55-59 | 2,464 | 24.13% |
| 60-64 | 2,051 | 20.08% |
| 65-69 | 1,944 | 19.04% |
| 70+ | 509 | 4.98% |
| Missing | 1 | 0.01% |
|  |  |  |
| **Ethnicity (of subject screened**^b^**)** |  |  |
| White – British or Irish or other | 6,455 | 63.21% |
| Asian – British Indian or Pakistani or Bangladeshi or other | 643 | 6.30% |
| Black – British or Caribbean or other | 892 | 8.73% |
| Black – African | 189 | 1.85% |
| Mixed White and Black, White and Asian or any other mixed | 119 | 1.17% |
| Chinese | 319 | 3.12% |
| Missing or not reported | 1,595 | 15.62% |
|  |  |  |
| **Breast Volumetric measurements**^c^  Median Breast volume, cm^3^  Median Dense volume cm^3^  Median %Mammographic Density | **Median**  758  49.9  6.4% | **IQR**  479-1,145  36.7-69.8  4.5%-10.2% |
|  |  |  |
| **Imaging acquisition parameters average across MLO and CC views**^c^  Mean compression force applied, N  Mean paddle tilt angle, degrees positive from horizontal ^d^  Mean pressure, kPa  **Imaging outcome estimates average across MLO and CC views**  Manufacturers mean glandular dose, mGy ^e^  Mean breast thickness, mm | **Mean**  8.26  2.52  8.48  1.33  55.8 | **SD**  2.11  0.99  3.52  0.36  12.4 |
|  |  |  |

^a^ A non-standard screening examination had <4 or >4 images taken, only screening appointments are included, excluded 22 images taken on non-Hologic systems and 648 screens excluded where women were known to have previous cancer.

^b^ Count for each screening examination (subjects may have more than one examination over the study period).

^c^ Calculated from the average value from the images available. Where > 4 images taken each image may only include part of the breast and the automated estimating algorithm is not able to make reliable overall volumetric estimates in these conditions.

^d^ Mean paddle tilt from horizontal (where paddle tilt >=0)

^e^ Manufacturers mean glandular dose as recorded in DICOM header
