# Supplementary material for: To what extent are objectively measured mammographic imaging techniques associated with compression outcomes
Source: Br J Radiol. 2023 Apr 20;96(1146):20230089. doi: 10.1259/bjr.20230089 (PMC10230394; doi:10.1259/bjr.20230089)
Supplement: Supplementary Figure 1. [file bjr.20230089.suppl-01.pptx]

## Slide 1
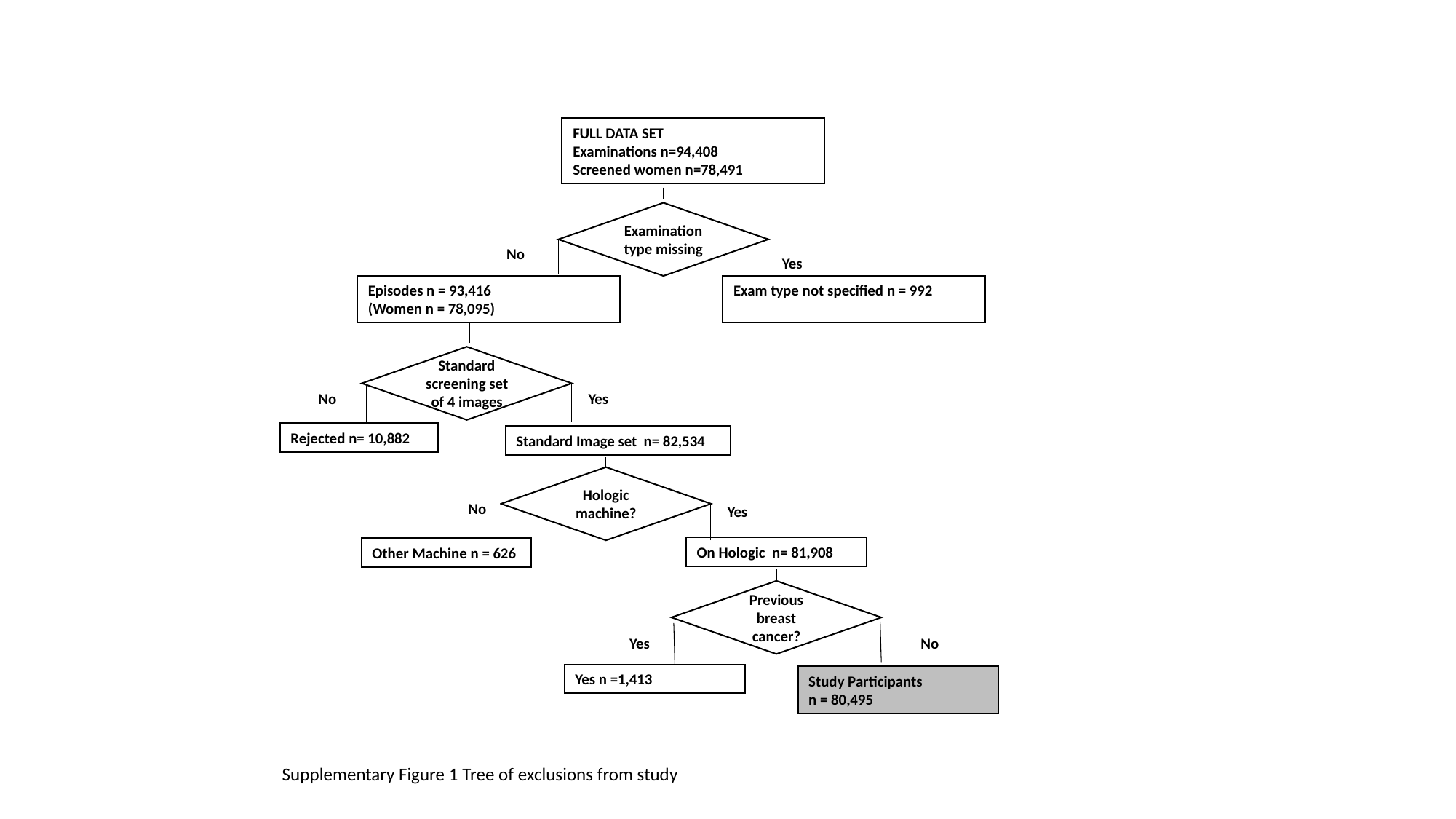

FULL DATA SET
Examinations n=94,408
Screened women n=78,491
Examination type missing
No
Yes
Episodes n = 93,416
(Women n = 78,095)
Exam type not specified n = 992
Standard screening set of 4 images
No
Yes
Rejected n= 10,882
Standard Image set n= 82,534
Hologic machine?
No
Yes
On Hologic n= 81,908
Other Machine n = 626
Previous breast cancer?
Yes
No
Yes n =1,413
Study Participants
n = 80,495
Supplementary Figure 1 Tree of exclusions from study
