# Supplementary material for: To what extent are objectively measured mammographic imaging techniques associated with compression outcomes
Source: Br J Radiol. 2023 Apr 20;96(1146):20230089. doi: 10.1259/bjr.20230089 (PMC10230394; doi:10.1259/bjr.20230089)
Supplement: Supplementary Figure 2. [file bjr.20230089.suppl-02.pptx]

## Slide 1
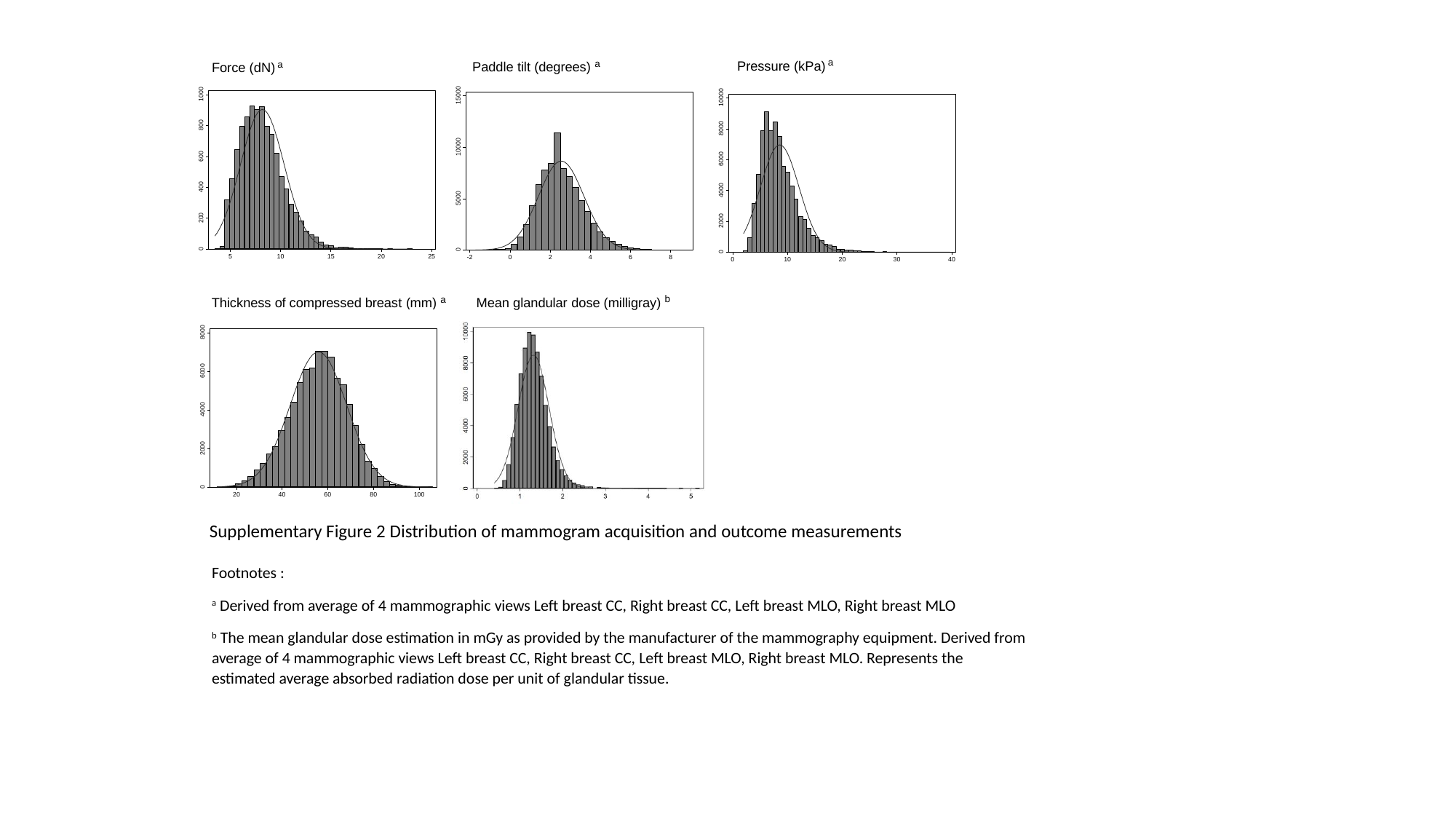

Supplementary Figure 2 Distribution of mammogram acquisition and outcome measurements
Footnotes :
a Derived from average of 4 mammographic views Left breast CC, Right breast CC, Left breast MLO, Right breast MLO
b The mean glandular dose estimation in mGy as provided by the manufacturer of the mammography equipment. Derived from average of 4 mammographic views Left breast CC, Right breast CC, Left breast MLO, Right breast MLO. Represents the estimated average absorbed radiation dose per unit of glandular tissue.
