# Supplementary material for: To what extent are objectively measured mammographic imaging techniques associated with compression outcomes
Source: Br J Radiol. 2023 Apr 20;96(1146):20230089. doi: 10.1259/bjr.20230089 (PMC10230394; doi:10.1259/bjr.20230089)
Supplement: Supplementary Figure 3. [file bjr.20230089.suppl-03.pptx]

## Slide 1
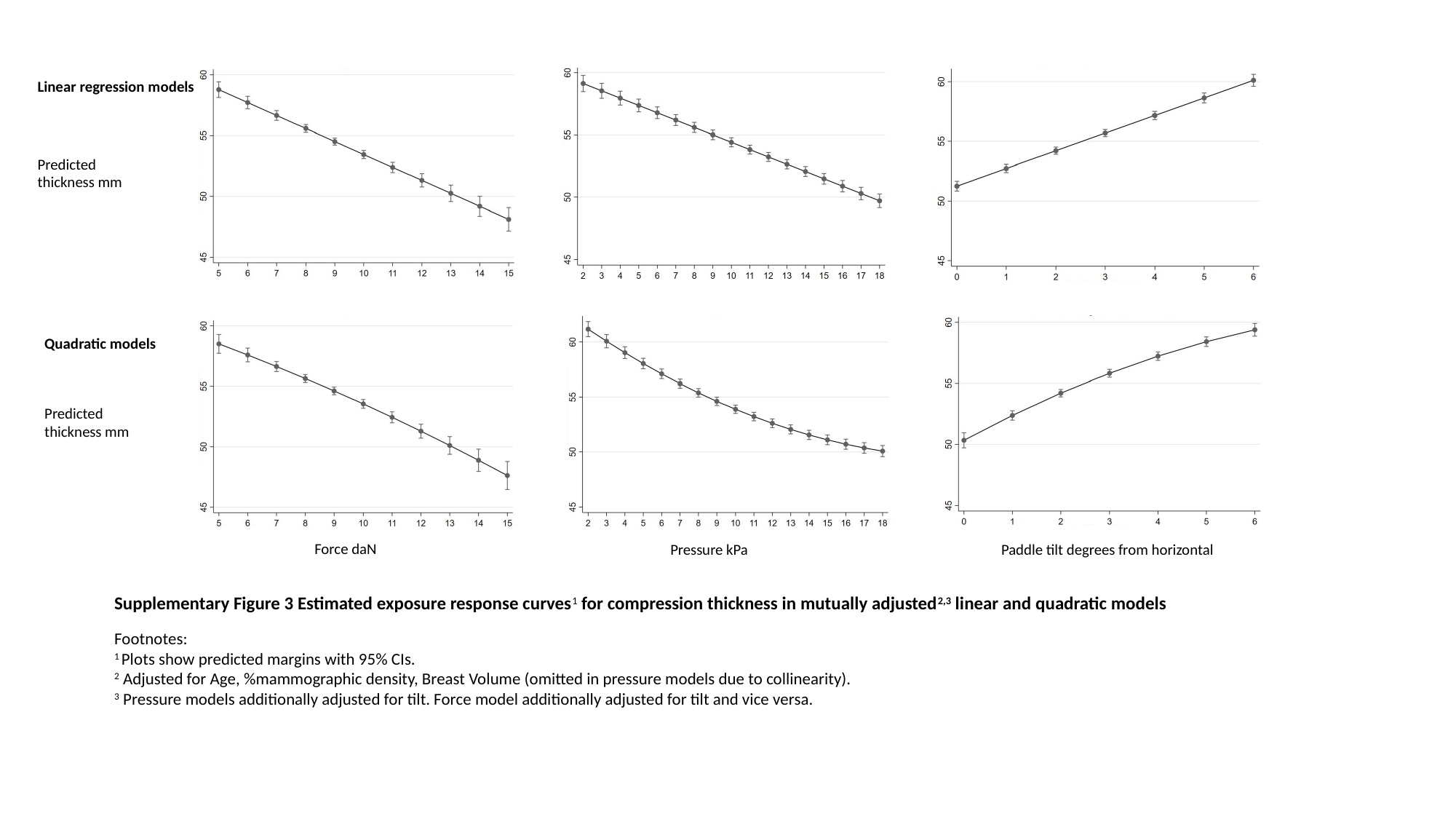

Linear regression models
Predicted thickness mm
Quadratic models
Predicted thickness mm
Force daN
Paddle tilt degrees from horizontal
Pressure kPa
Supplementary Figure 3 Estimated exposure response curves1 for compression thickness in mutually adjusted2,3 linear and quadratic models
Footnotes:
1 Plots show predicted margins with 95% CIs.
2 Adjusted for Age, %mammographic density, Breast Volume (omitted in pressure models due to collinearity).
3 Pressure models additionally adjusted for tilt. Force model additionally adjusted for tilt and vice versa.
